# Endothelial SARS-CoV-2 infection is not the underlying cause of COVID-19-associated vascular pathology in mice

**DOI:** 10.3389/fcvm.2023.1266276

**Published:** 2023-09-26

**Authors:** Siqi Gao, Alan T. Tang, Min Wang, David W. Buchholz, Brian Imbiakha, Jisheng Yang, Xiaowen Chen, Peter Hewins, Patricia Mericko-Ishizuka, N. Adrian Leu, Stephanie Sterling, Avery August, Kellie A. Jurado, Edward E. Morrisey, Hector Aguilar-Carreno, Mark L. Kahn

**Affiliations:** ^1^ Department of Medicine and Cardiovascular Institute, University of Pennsylvania, Philadelphia, PA, United States; ^2^ Department of Microbiology and Immunology, College of Veterinary Medicine, Cornell University, Ithaca, NY, United States; ^3^ Department of Microbiology, University of Pennsylvania Perelman School of Medicine, Philadelphia, PA, United States; ^4^ Department of Biomedical Sciences, School of Veterinary Medicine, University of Pennsylvania, Philadelphia, PA, United States; ^5^ Penn-CHOP Lung Biology Institute, Perelman School of Medicine, University of Pennsylvania, Philadelphia, PA, United States

**Keywords:** SARS-CoV-2, endothelial cell, COVID-19, vascular, hACE2

## Abstract

Endothelial damage and vascular pathology have been recognized as major features of COVID-19 since the beginning of the pandemic. Two main theories regarding how severe acute respiratory syndrome coronavirus 2 (SARS-CoV-2) damages endothelial cells and causes vascular pathology have been proposed: direct viral infection of endothelial cells or indirect damage mediated by circulating inflammatory molecules and immune mechanisms. However, these proposed mechanisms remain largely untested *in vivo*. In the present study, we utilized a set of new mouse genetic tools developed in our lab to test both the necessity and sufficiency of endothelial human angiotensin-converting enzyme 2 (hACE2) in COVID-19 pathogenesis. Our results demonstrate that endothelial ACE2 and direct infection of vascular endothelial cells do not contribute significantly to the diverse vascular pathology associated with COVID-19.

## Introduction

The most common clinical feature reported in patients with COVID-19 is respiratory symptoms (1, 2). In addition to primarily causing pulmonary symptoms, COVID-19 disease is accompanied by vascular pathology, endothelial damage, and vascular coagulopathy (3–5). Reports emerged around the world confirming a disproportionate prevalence of abnormal thrombotic events and vascular pathology in patients with COVID-19, even in those not in intensive care units (6–14). Theories regarding the mechanism of vascular disease observed in patients with COVID-19 have been proposed, including direct infection of endothelial cells and systemic inflammatory responses (15–22). However, these hypotheses remain largely untested, and the cellular basis of vascular pathology remains controversial. In this study, we used a set of new mouse genetic tools (23) to rigorously test endothelial contribution to COVID-19-associated vascular pathology.

## Materials and methods

### Mice

*hACE2^fl/y^* mice and LSL-hACE2^+/0^ mice have been generated through CRISPR/Cas9-assisted mouse embryonic stem cell targeting and have been described (23). Briefly, for loss of function mouse line, the human ACE2 cDNA sequence was inserted after the ATG-start codon of mouse *Ace2* in exon2, flanking the polyA cassette with loxP sites, to achieve Cre-mediated cell type-specific deletion of ACE2. For gain of function mouse line, the human ACE2 cDNA sequence was targeted to Rosa26 locus with Lox-STOP-Lox cassette to permit tissue-specific gain of expression of ACE2. Tie2-Cre transgenic mice have been used for tissue-specific drivers as previously described (24). All mice were maintained on a mixed genetic background, including C57BL/8 and other strains, at the University of Pennsylvania animal facility. Mice were genotyped by PCR as described (23). The number of male mice used in each experiment ranged from three to nine.

### Viral inoculation and tissue harvest

Viral inoculations were performed as described previously (23). Briefly, mice were anesthetized with isoflurane and then intranasally infected with SARS-CoV-2 (Isolate USA-WA1/2020; BEI resources: NR-52281) that was obtained from BEI Resource. Mice were monitored and weighed daily, then euthanized at a humane endpoint when they lost 20% of their starting weights. Mice studies were combined results from Penn ABSL3 laboratory and Cornell ABSL3 laboratory in accordance with protocols approved by the IACUC at the University of Pennsylvania and Cornell University. For tissue harvest, mice were euthanized with ketamine/xylazine. Lungs were gently inflated with PBS infusion via trachea cannulation. Then lungs were fixed in 4% paraformaldehyde with a minimum of 72 h to ensure viral inactivation. Tissues were removed from the animal BSL3 facility, followed by ethanol dehydration and embedding in paraffin blocks for histology. Hematoxylin and eosin staining was performed on paraffin sections.

### Immunofluorescence staining and analysis

Immunohistochemistry staining was performed as previously described (23) with control and experimental samples on the same slide and under identical staining conditions. Primary antibodies were as follows: pan-ACE2 (goat, 1:1,000, R&D AF933), hACE2 (rabbit, 1:200, Abcam ab108209), SARS-CoV-2 nucleocapsid (rabbit, 1:500, Rockland 200-401-A50), ICAM-1 (rabbit, 1:500, Abcam ab179707), vWF (rabbit 1:1,000, Novus Biologicals NB600-586), and PECAM (goat 1:500, R&D AF3628). Fluorescence-conjugated Alexa Fluor secondary antibodies were used (1:500, Invitrogen) according to the primary antibody species and counterstained with DAPI (1:1,000). ICAM1 and vWF fluorescence intensity were calculated by integrated fluorescence intensity. All images were analyzed using ImageJ/FIJI software.

### Statistics

Mice were inoculated with SARS-CoV-2 in a blinded fashion without knowledge of genotypes, and infections were performed in two different ABSL-3 facilities with independent experimenters. Statistical tests used to determine significance are described in the figure legends. GraphPad Prism 9.5.1 was used to generate graphs and statistical analyses. Survival curve statistics were performed with log-rank Mantel-Cox tests. All *t*-tests performed were two-tailed.

## Results and discussion

**Figure 1:**
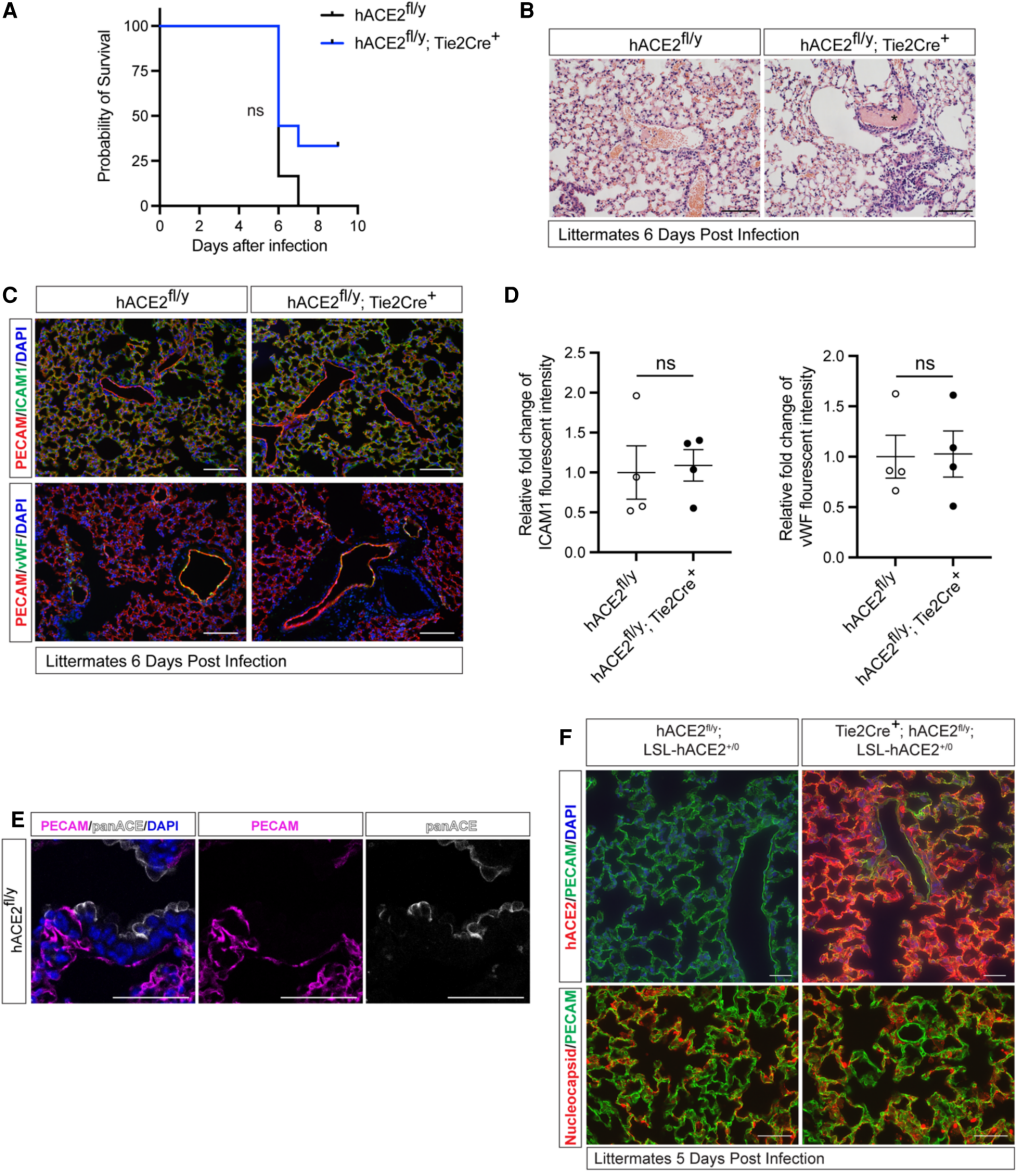
Loss or gain of endothelial hACE2 does not alter SARS-CoV2 infection. (**A**) Survival of *hACE2^fl/y^* and *hACE2^fl/y^; Tie2-Cre^+^* male mice (12 to 16-week-old males) after infection with 10^5^ PFU of SARS-CoV-2 via intranasal administration. This viral inoculation method was used in all experiments. *n* = 6 (*hACE2^fl/y^*) and 9 (*hACE2^fl/y^; Tie2-Cre^+^*); ns, non-significant; data are from two independent experiments. (**B**) H&E staining of *hACE2^fl/y^* and *hACE2^fl/y^; Tie2-Cre^+^* lung tissue 6 days after infection. The asterisk indicates intravascular thrombosis. Scale bars: 100 μm. (**C**) Immunofluorescent staining of the lung from *hACE2^fl/y^* and *hACE2^fl/y^; Tie2-Cre^+^* mice with antibodies against ICAM1 or vWF (green), and PECAM (red). Images are representative of four animals per genotype. Scale bars: 100 μm. (**D**) Quantification of ICAM1 and vWF fluorescent intensity. The error bars represent mean ± s.d; statistical analyses were performed using an unpaired two-tailed *t*-test; ns, non-significant. (**E**) Immunofluorescent staining of *hACE2^fl/y^* lung tissue using pan-ACE2 antibodies (grey) that recognize both hACE2 and mACE2 proteins and co-stained with PECAM (magenta). Images are representative of three animals. Scale bars 50 μm. (**F**) Immunofluorescent staining of the lung from *hACE2^fl/y^;* LSL-hACE2^+/0^ and Tie2Cre^+^; *hACE2^fl/y^;* LSL-hACE2^+/0^ mice is performed using anti-hACE2 antibody or anti-SARS-CoV-2 nucleocapsid (red) and costained with PECAM (green) 5 days after infection with SARS-CoV-2. The *hACE2^fl/y^* allele enables these mice to be productively infected intranasally. Representative of three animals per genotype. Scale bars 100 μm.

Cellular expression of ACE2 is indispensable for SARS-CoV-2 infection in pneumocytes (25, 26), but SARS-CoV-2 is unable to bind mouse ACE2. To determine if endothelial cells directly contribute to lethal infection, we generated animals that express human ACE2 (hACE2) from the mouse Ace2 locus in a manner that enables cell-specific loss of hACE2 using Cre recombinase (*hACE2^fl/y^* mice) (23). We crossed *hACE2^fl/y^* mice onto a *Tie2-Cre* transgenic mouse line that drives Cre expression in endothelial cells (ECs) to generate mice that express hACE2 in all cells except vascular ECs. *hACE2^fl/y^; Tie2-Cre^+^* mice and control littermates were exposed to 10^5^ PFU of SARS-CoV-2 virus via nasal inhalation. *hACE2^fl/y^; Tie2-Cre^+^* mice showed no significant difference in survival after exposure to SARS-CoV-2 compared with the littermate controls (Figure 1A). Histological analysis revealed the presence of alveolar infiltrates and pulmonary vascular thrombi in the lungs of infected *hACE2^fl/y^; Tie2-Cre^+^* mice that were indistinguishable from findings observed in control *hACE2^fl/y^* mice (Figure 1B). Histological analysis using hematoxylin-eosin staining of tissue sections from the small intestine, kidney, liver, and heart also failed to identify any vascular pathology (Supplementary Figure S1). Next, we evaluated the expression of inflammation-induced protein intracellular adhesion marker 1 (ICAM1) and the pro-coagulant, inflammation-induced protein Von Willebrand factor (vWF) in the mice following the SARS-CoV-2 infection, given both ICAM1 and vWF have been closely associated with COVID-19 induced vascular damage (27, 28). Expression of ICAM1 and vWF were also similar in the lung capillary endothelial cells of SARS-CoV-2-infected *hACE2^fl/y^* and *hACE2^fl/y^; Tie2-Cre^+^* mice (Figures 1C,D).

The studies described above suggested that endothelial cell infection is not required for vascular COVID-19 pathology when hACE2 is expressed at endogenous levels. In fact, immunostaining of lung sections using anti-ACE2 antibodies was able to detect ACE2 expression in epithelial but not endothelial cells (23) (Figure 1E). To more rigorously test the role of endothelial hACE2, we next crossed *Tie2-Cre* onto a recently described Cre-activated gain of function hACE2 allele (loxP-stop-loxP-hACE2 or LSL-hACE2^+/0^) (23) to over-express hACE2 in vascular endothelial cells. *Tie2-Cre*;LSL-hACE2^+/0^ animals exhibited very high endothelial-specific expression of hACE2, assessed by immunostaining of tissue sections compared with *hACE2^fl/y^* mice (Figure 1F). To ensure that *Tie2-Cre*;LSL-hACE2^+/0^ animals would be productively infected following SARS-CoV-2 exposure, we generated *Tie2-Cre*;LSL-hACE2^+/0^;*hACE2^fl/y^* animals that support robust infection of the nasal and respiratory epithelium (23) (Figure 1F). Despite high levels of endothelial hACE2 expression, we failed to detect nucleocapsid protein that colocalized with PECAM^+^ endothelial cells following nasal SARS-CoV-2 infection (Figure 1F). In contrast, we have previously shown that this gain of function allele is sufficient to drive hACE2 expression and support SARS-CoV-2 infection in both neuronal cells and lung epithelial cells (23). These studies support the conclusion that SARS-CoV-2 does not confer endothelial cell damage and vascular thrombosis through direct viral infection of those cells. They further demonstrate that the levels of circulating virus are too low to infect even endothelial cells that express very high levels of hACE2, and therefore that most COVID-19 pathology arises due to aerosol infection of the nasal and pulmonary epithelium.

It has been debated whether direct viral infection of endothelial cells or indirect damage from systematic inflammation underlie COVID-19-associated vascular pathology (3). Our murine vascular endothelial loss and gain of function studies reported here provide strong *in vivo* evidence that endothelial ACE2 and direct infection of vascular endothelial cells do not contribute significantly to the diverse vascular pathology associated with COVID-19. These findings are consistent with previously reported *in vitro* studies that showed human endothelial cells are not readily infected by SARS-CoV-2 (21). Together with our recently reported studies, these findings strongly support a mechanism in which SARS-CoV-2 infection of nasal epithelial and neuronal cells stimulates a powerful inflammatory response that is the cause of COVID-19 vascular pathology.

## Limitations of the study

In the present study, we utilized both loss of function and gain of function hACE2 mouse lines and demonstrated that direct endothelial viral infection does not contribute to COVID-19-associated vascular pathology. Future studies are needed to define the cytokines that likely drive secondary vascular inflammation and thrombosis and to understand the molecular mechanism by which systemic inflammation damages endothelium following SARS-CoV-2 infection. We used the original isolate SARS-CoV-2 USA-WA1/2020 strain in our study because that isolate is the best characterized regarding vascular complication. Omicron BA.1 variant failed to confer lethal disease and associated vascular phenotypes in our mouse models (23). Future studies testing the impact of other variants on the vascular system will be needed. We performed our studies on male mice due to the *Ace2* allele being located on the X chromosome, enabling a straightforward comparison. Mouse models are not humans, and our mouse model *hACE2^fl/y^* expresses a higher level of hACE2 as previously reported (23). Thus there are likely to be differences in pathogenic mechanisms identified using our model compared to human studies. However, this difference should bias toward rather than against a direct endothelial infection mechanism and it does not weaken our negative conclusions. Future studies looking at longer-term vascular events in mice with lower levels of hACE2 expression will be needed to address non-acute mechanisms of COVID-19-related cardiovascular disease.

## Data Availability

The original contributions presented in the study are included in the article/**Supplementary Material**, further inquiries can be directed to the corresponding author.
