# Supplementary material for: Endothelial SARS-CoV-2 infection is not the underlying cause of COVID-19-associated vascular pathology in mice
Source: Front Cardiovasc Med. 2023 Sep 26;10:1266276. doi: 10.3389/fcvm.2023.1266276 (PMC10562591; doi:10.3389/fcvm.2023.1266276)
Supplement: Supplementary file 1 [file Image1.pdf]

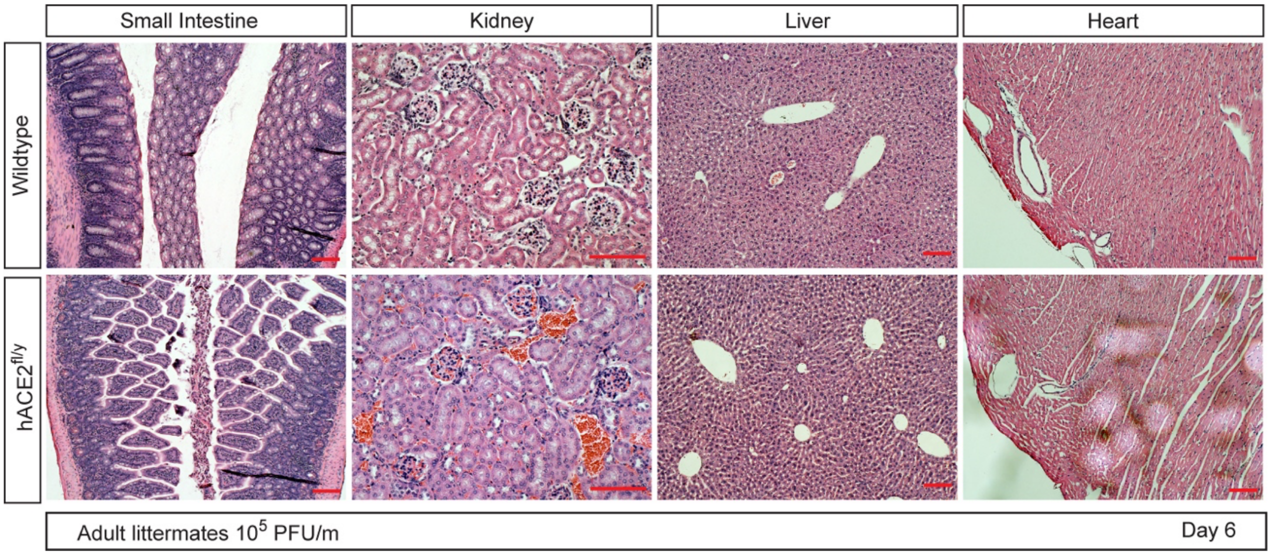

Supplementary Figure 1. H&E analysis of small intestine, kidney, liver, and heart from *hACE2*$^{fl/y}$ mice 6 days after 10^5 PFU of SARS-CoV-2 infection via intranasal administration. Scale bars, 100 µm.
